# Comparison of bioelectrical impedance analysis and dual-energy X-ray absorptiometry for the diagnosis of sarcopenia in the older adults with metabolic syndrome: equipment-specific equation development

**DOI:** 10.1007/s40520-024-02898-1

**Published:** 2024-12-27

**Authors:** Younji Kim, Jaewon Beom, Sang Yoon Lee, Hak Chul Jang, Keewon Kim, Miji Kim, Ga Yang Shim, Chang Won Won, Jae-Young Lim

**Affiliations:** 1 Department of Rehabilitation Medicine, School of Medicine, Ewha Woman’s University Seoul Hospital, Seoul, Republic of Korea; 2 Department of Rehabilitation Medicine, Seoul National University College of Medicine, Seoul National University Bundang Hospital, 82, Gumi-ro 173beon-gil, Bundang-gu, Seongnam-si, Gyeonggi-do 13620 Republic of Korea; 3 Department of Rehabilitation Medicine, Seoul National University College of Medicine, SMG-SNU Boramae Medical Center, Seoul, Republic of Korea; 4 Department of Internal Medicine, Seoul National University College of Medicine, Seoul National University Bundang Hospital, Seongnam, Republic of Korea; 5 Department of Rehabilitation Medicine, Seoul National University College of Medicine, Seoul National University Hospital, Jongno-gu, Seongnam, Republic of Korea; 6 Department of Biomedical Science and Technology, College of Medicine, East-West Medical Research Institute, Kyung Hee University, Seoul, Republic of Korea; 7 Department of Physical Medicine & Rehabilitation, College of Medicine, Kyung Hee University, Seoul, Republic of Korea; 8 Department of Family Medicine, College of Medicine, Kyung Hee University, Kyung Hee University Medical Center, Seoul, Republic of Korea; 9 Institute on Aging, Seoul National University, Seoul, Republic of Korea

**Keywords:** Sarcopenia, Diagnostic equipment, Bioelectrical impedance, Dual-energy X-ray absorptiometry, Metabolic syndrome

## Abstract

**Objective:**

Metabolic syndrome (MetS) and sarcopenia together pose significant health risks, increasing frailty, falls, and fractures in older adults. This study compared muscle mass measurements obtained using two different dual-energy X-ray absorptiometry (DXA) machines and bioelectrical impedance analysis (BIA), and evaluated the accuracy of these measurements in these older adults.

**Methods:**

In this prospective multicenter cohort study, patients aged ≥ 65 years with MetS had their muscle mass assessed using both BIA and DXA. Two DXA devices, Hologic Horizon and GE Lunar Prodigy, were used as clinical standards for sarcopenia diagnosis. Statistical analyses generated equations for transforming BIA results to match those from DXA, enhancing comparability.

**Results:**

Participants had a mean age of 73.2 ± 5.3 years. The mean appendicular skeletal muscle mass (ASM) measured by BIA and DXA was 19.7 ± 3.1 kg (BIA) and 18.1 ± 2.9 kg (DXA) for males, and 13.7 ± 2.2 kg (BIA) and 12.6 ± 1.8 kg (DXA) for females. Device-specific equations were developed to estimate DXA-measured ASM based on BIA results. These equations are presented for all participants and for each DXA device, highlighting significant differences in prediction models between the two DXA machines.

**Conclusion:**

The study developed device-specific equations for sarcopenia diagnosis in older adults with MetS, highlighting substantial differences between Hologic and GE Lunar devices. While BIA may offer a more accessible alternative to DXA, the variation in prediction formulas underscores the need for standardized equipment to ensure consistency in sarcopenia diagnosis.

**Supplementary Information:**

The online version contains supplementary material available at 10.1007/s40520-024-02898-1.

## Introduction

The global growth in the population increase in size of the older adults has led to an increase in age-related diseases, particularly sarcopenia and metabolic syndrome (MetS) [1]. Sarcopenia is defined by a decline in muscle mass, strength, and function [2], and MetS is characterized by obesity and insulin resistance [3]. Both conditions significantly contribute to increased frailty and morbidity among older adults [3]. Their concurrent presence exacerbates health risks, negatively impacting the quality of life and independence of older adults [4, 5].

Recent guidelines for diagnosing sarcopenia recommend a comprehensive, multi-step approach, emphasizing body composition assessment with a particular focus on quantifying muscle mass in the final stage [6, 7]. Common tools for measuring muscle mass include dual-energy X-ray absorptiometry (DXA), bioelectrical impedance analysis (BIA), magnetic resonance imaging, and computed tomography [8, 9]. Of these, BIA and DXA are the most frequently used [6, 7]. However, the accuracy of muscle mass measurements is often compromised by inter-measurement and inter-device variability, along with a lack of standardization among these assessment tools. Such variability can significantly affect the precision of sarcopenia diagnosis.

BIA is a rapid, non-invasive, and cost-effective method for estimating muscle mass and body composition. It measures impedance to electrical current flow through different body tissues using electrodes applied to the skin, exploiting the varying conductivity of these tissues [10, 11]. However, the accuracy of BIA is somewhat compromised by several factors, including variations in equipment, operator skill, subject-specific characteristics, and environmental influences. These factors can make BIA results less reliable compared to those obtained using more standardized methods.

DXA provides an accurate assessment of body composition using X-rays and is considered the gold standard in sarcopenia research due to its accessibility, cost-effectiveness, low radiation exposure, rapid scanning time, and comprehensive data from whole-body scans [9, 12]. However, significant variations in measurements have been observed across different DXA machines, such as the Hologic Horizon DXA system (Hologic Inc., Bedford, MA, USA), the GE Lunar DXA machine (GE Healthcare Lunar, Madison, MI, USA), and Norland systems (Norland Corp., Fort Atkinson, WI, USA) [13, 14]. These discrepancies can be attributed to differences in calibration, software algorithms, and scanning protocols among manufacturers, which may result in inconsistent results when comparing data from different DXA systems [14–16]. Therefore, careful interpretation of DXA results is essential, particularly in longitudinal studies or comparative research involving various DXA devices.

Both BIA and DXA offer distinct advantages and are widely utilized in clinical and research settings. However, several challenges persist: how well do these assessment methods agree, and what are the implications for measuring muscle mass in the older adults? Additionally, how consistent are measurements between devices, even when using the same method? Although existing studies have examined the validity and reliability of BIA and DXA for muscle mass assessment [15, 17–19], a comprehensive comparison—particularly focusing on different DXA devices within the older adults with MetS—remains to be conducted.

Given these conditions, this study compared body composition measurements obtained from BIA and DXA within the older adults with MetS. Furthermore, we assessed the concordance and correlation between these methods. In response to the observed differences, we developed device-specific equations to approximate DXA values from BIA measurements for each DXA machine used. This approach seeks to address and mitigate measurement discrepancies, providing practical solutions to enhance accuracy and consistency in muscle mass assessment.

## Methods

### Participants

This study involved a secondary data analysis derived from a multicenter, prospective cohort study aimed at comparing body composition measurements obtained via BIA and DXA. The original trial was prospectively registered on ClinicalTrials.gov (NCT04948736) before participant recruitment began. The research focused on community-dwelling older adults aged 65–90 years. We excluded individuals younger than 65 years or older than 90 years, those without MetS, those who did not complete both BIA and DXA testing, and those who declined to participate. The study was conducted from January 2022 to October 2023, including 234 patients diagnosed with MetS.

The comprehensive data analysis included baseline demographic information as well as detailed body composition measurements obtained through both BIA and DXA methodologies. Each study participant underwent both BIA and DXA procedures, ensuring a complete dataset for comparative analysis. Ethical approval for the study was granted by the Institutional Review Board of Seoul National University Bundang Hospital (no. B-2010-645-005). In accordance with ethical guidelines, all participants provided informed consent before being included in the study.

### Definition of MetS and low appendicular skeletal muscle mass (ASM)

MetS was defined according to the following criteria [20], where at least three of the following conditions had to be present: waist circumference ≥ 90 cm in males and ≥ 85 cm in females (Asian cut-off); systolic blood pressure ≥ 130 mm Hg, diastolic blood pressure ≥ 85 mm Hg, or treatment for hypertension; fasting blood glucose ≥ 5.6 mmol/L (100 mg/dL) or treatment for elevated glucose levels; triglycerides ≥ 1.7 mmol/L (150 mg/dL) or treatment for hypertriglyceridemia; and high-density lipoprotein cholesterol (HDL-C) < 1.0 mmol/L (40 mg/dL) in males and < 1.3 mmol/L (50 mg/dL) in females.

Low ASM was defined according to the criteria proposed by the Asian Working Group for Sarcopenia 2019 [6]. For DXA, a low ASM index (ASMI, ASM/height²) was defined as < 7.0 kg/m² in males and < 5.4 kg/m² in females. For BIA, a low ASMI was defined as < 7.0 kg/m² in males and < 5.7 kg/m² in females.

### High-frequency BIA

Muscle mass was measured using multi-frequency BIA with either the Inbody770 or Inbody970 device (Biospace, Seoul, Korea). Testing was conducted after a 12-h fasting period. Participants were tested while wearing lightweight clothing and without jewelry, metals, or shoes. The BIA devices use eight tactile electrodes: four placed on the hands (palm and thumb) and four on the feet. Results, including total and regional (arms, legs, and trunk) skeletal muscle mass and fat mass, were displayed on the screen and printed. The skeletal muscle mass (kg) of the arms and legs, as shown in the evaluation report, was recorded as the ASM value.

### DXA

For DXA assessments, participants were clothed in the lightweight gowns and pants provided. Sequential BIA and DXA examinations were conducted on the same day in this multi-center study, following each manufacturer’s standard protocols. Depending on the participating institution, the DXA device used was either a GE Lunar DXA or a Hologic Horizon DXA system. These machines measured total body, regional body fat mass, body fat percentage, and ASM. The GE Lunar Prodigy scanner automatically selected the scan mode, while the Hologic Horizon W scanner utilized the array mode for body composition measurements, in accordance with respective manufacturer protocols. Data analysis was performed using GE Lunar DXA Encore software version 13.60 or Hologic APEX software version 5.6.0.4. During scans, participants were positioned with their hands placed laterally and feet rotated medially at 15°.

### Statistical analysis

All statistical analyses were conducted using SPSS for Windows (version 27.0; IBM Corp., Armonk, NY, USA). Data are presented as mean ± standard deviation or as numerical counts with corresponding percentages. To compare baseline characteristics between male and female participants, an independent-sample t-test was used for continuous variables, whereas the Pearson chi-square test or Fisher’s exact test (for expected cell counts < 5) was used for categorical variables. Pearson correlations were computed to examine the relationship between BIA and DXA muscle measurements. Multivariable regression analysis was employed to assess the potential influence of anthropometric parameters such as age, sex, height, and weight. Stepwise linear regression was used to develop four prediction equations based on BIA measurements. The Bland–Altman method was applied to evaluate the concordance between muscle mass measurements obtained from BIA and DXA. *P* < 0.05 was considered indicative of statistical significance.

## Results

**Fig. 1:**
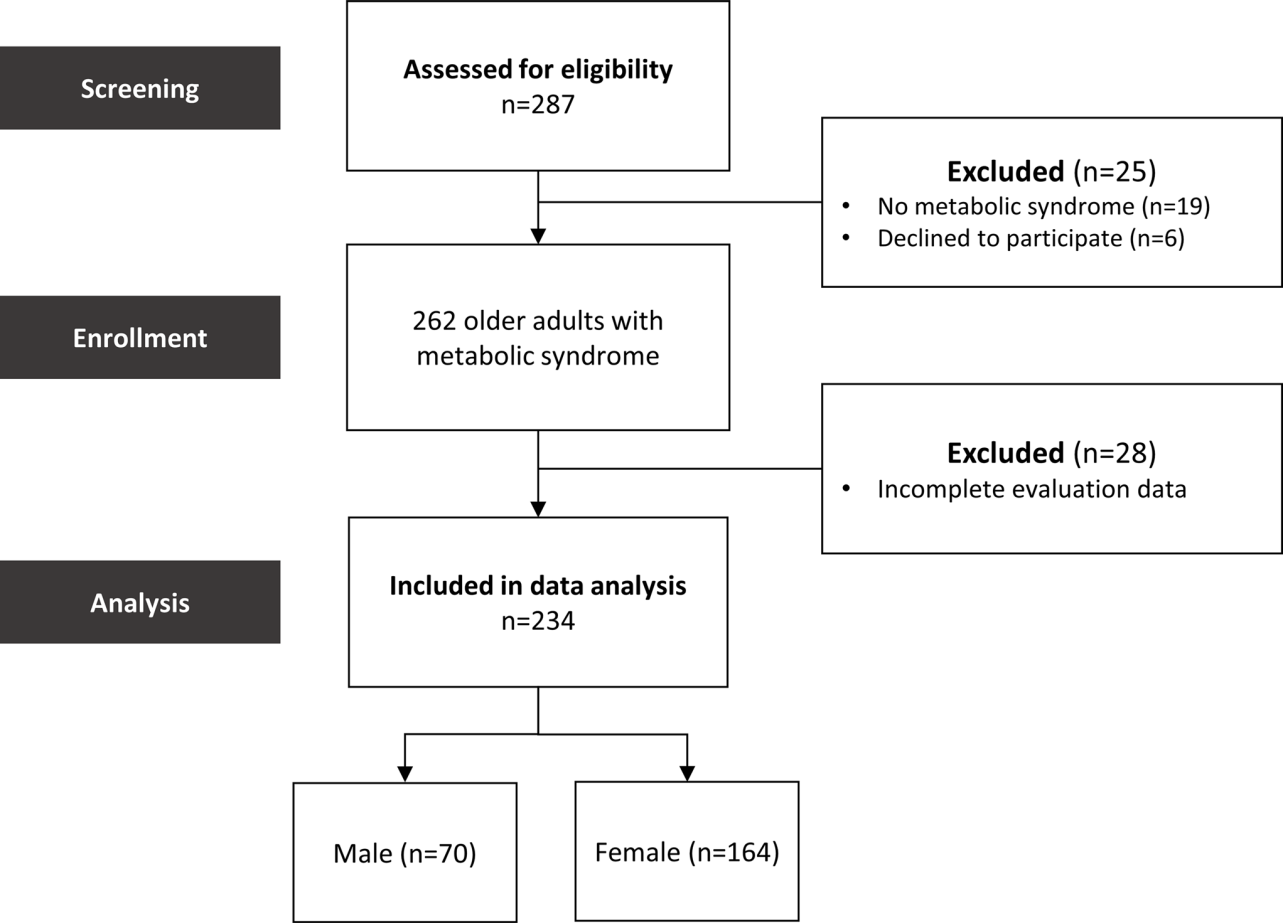
Flowchart of patients’ enrollment process

In our study, we recruited older adults aged 65–90 years with MetS. After screening, 287 individuals were deemed eligible for participation, of whom 234 chose to take part. All participants underwent both BIA and DXA assessments (using the Hologic Horizon or GE Lunar DXA devices). Figure 1 presents the participant characteristics. The final cohort included 234 older adults (70 males and 164 females) for the comparison of muscle mass measurements between the two tests.

### General characteristics of study participants

Table 1 presents the descriptive characteristics of the study participants for the two DXA machines. The mean age of the participants was 73.2 years. Among the 70 men, 37 had their muscle mass evaluated with the Hologic Horizon, and 33 with the GE Lunar DXA machine. There were no significant differences in age, height, weight, or body mass index (BMI) between the men tested on the two devices. Among the 164 women, 75 were evaluated using the Hologic Horizon, and 89 with the GE Lunar DXA. Although there were no significant differences in age between the two groups of women, those tested with the GE Lunar DXA had lower height, weight, and BMI values compared to those tested with the Hologic Horizon.

**Table 1:** Descriptive characteristics of study participants

Variables	Total (*n* = 234)	Male (*n* = 70)			Female (*n* = 164)		
		Hologic (*n* = 37)	GE Lunar (*n* = 33)	*p*-value	Hologic (*n* = 75)	GE Lunar (*n* = 89)	*p*-value
Age (yr)	73.2 ± 5.3	73.5 ± 5.1	73.7 ± 6.2	0.910	72.5 ± 5.0	73.4 ± 5.3	0.280
Height (cm)	156.8 ± 8.0	166.6 ± 5.7	164.7 ± 5.7	0.158	154.2 ± 4.7	152.1 ± 6.0	0.015
Weight (kg)	57.3 ± 8.7	65.4 ± 8.6	61.5 ± 8.5	0.063	57.0 ± 6.7	52.6 ± 7.6	< 0.001
BMI (kg/m^2^)	23.3 ± 2.9	23.5 ± 2.7	22.7 ± 2.5	0.155	24.0 ± 2.8	22.8 ± 3.1	0.008
BIA							
PBF (%)	31.4 ± 7.1	27.4 ± 4.7	23.5 ± 5.2	0.002	34.7 ± 5.7	33.1 ± 6.8	0.102
ASM (kg)	15.5 ± 3.7	20.0 ± 3.0	19.4 ± 3.2	0.374	14.4 ± 1.9	13.1 ± 2.2	< 0.001
Low ASMI	106 (45.3)	16 (43.2)	18 (54.5)	0.345	20 (26.7)	52 (58.4)	< 0.001
DXA							
PBF (%)	35.8 ± 7.0	31.1 ± 4.2	26.9 ± 5.1	< 0.001	40.3 ± 5.2	37.3 ± 5.5	< 0.001
ASM (kg)	14.3 ± 3.3	17.6 ± 3.1	18.5 ± 2.7	0.193	12.5 ± 1.8	12.8 ± 1.8	0.305
Low ASMI	148 (63.2)	28 (75.7)	23 (69.7)	0.574	56 (74.7)	41 (46.1)	< 0.001

Values are mean ± standard deviation or the number of patients (%)

BMI, body mass index; BIA, Bioelectrical impedance analysis; PBF, percentage total body fat; ASM, appendicular skeletal muscle mass; ASMI, appendicular skeletal muscle mass index; DXA, dual-energy X-ray absorptiometry

### Muscle mass measurements: BIA vs. DXA

Bland–Altman plots were used to assess the agreement between the muscle mass measurements obtained via the BIA and DXA methods (Fig S1). Four plots depicted the differences versus the averages of the two methods for absolute ASM and ASM adjusted by height squared (ASM/ht²), BMI (ASM/BMI), and weight (ASM/wt). The mean differences (red lines) were small across all plots, indicating minimal average discrepancies between BIA and DXA measurements. The distribution of data points within the limits of agreement (green dashed lines) showed variability in the differences, with significant agreement seen for most values.

Table 2 compares muscle mass measurements obtained by BIA and DXA in both men and women. The muscle mass values measured by BIA were significantly higher than those measured by DXA devices for both men and women (*p* < 0.001). Significant differences were observed in ASM, percentage of body fat, and ASM indices, including ASM/ht², ASM/wt, and ASM/BMI (*p* < 0.001).

**Table 2:** Comparison of muscle mass measured by BIA and DXA according to sex

Variables	Male (*n* = 70)			Female (*n* = 164)		
	BIA	DXA	*p*-value	BIA	DXA	*p*-value
PBF (%)	25.6 ± 5.3	29.1 ± 5.1	< 0.001	33.9 ± 6.3	38.7 ± 5.6	< 0.001
ASM (kg)	19.7 ± 3.1	18.1 ± 2.9	< 0.001	13.7 ± 2.2	12.6 ± 1.8	< 0.001
ASM/height^2^	7.1 ± 0.8	6.6 ± 0.9	< 0.001	5.8 ± 0.7	5.4 ± 0.7	< 0.001
ASM/weight	0.310 ± 0.025	0.284 ± 0.033	< 0.001	0.252 ± 0.029	0.233 ± 0.030	< 0.001
ASM/BMI	0.853 ± 0.112	0.781 ± 0.111	< 0.001	0.593 ± 0.096	0.546 ± 0.082	< 0.001
Low ASMI	34 (48.6)	51 (72.9)	0.001	72 (43.9)	97 (59.1)	0.001

Values are mean ± standard deviation or the number of patients (%)

BMI, body mass index; BIA, Bioelectrical impedance analysis; PBF, percentage total body fat; ASM, appendicular skeletal muscle mass; DXA, dual-energy X-ray absorptiometry

### Predictive equations for muscle mass estimated using BIA

Predictive models were developed to estimate muscle mass measured by DXA based on BIA values (Table 3). For the entire cohort, the predictive model for DXA-estimated ASM was established as follows: DXA_ASM = 13.669 + 0.830 × BIA_ASM − 0.072 × Height − 1.349 × Sex, where sex was coded as 0 for males and 1 for females. This model showed a strong correlation with actual DXA_ASM measurements, with an R² of 0.853 and an adjusted R² of 0.851. A refined model of the 112 patients measured with the Hologic device demonstrated even greater accuracy (adjusted R² = 0.910): DXA_ASM = 10.869 + 1.073 × BIA_ASM − 0.089 × Height. For the 122 patients evaluated with the GE Lunar device, the model was as follows: DXA_ASM = 3.717 + 0.505 × BIA_ASM + 0.082 × Weight − 1.879 × Sex (adjusted R² = 0.892). These models exhibited strong predictive capabilities across the total participant pool and for each DXA device individually (adjusted R² = 0.851–0.910). Models for ASM/ht², ASM/BMI, and ASM/wt also demonstrated considerable predictive strength (adjusted R² = 0.644–0.906).

**Table 3:** The development of the prediction models for muscle mass by BIA

Predication model development equations			*R*²	adjusted *R*²
Total (*n* = 234)	DXA_ASM	= 13.669 + 0.830*BIA_ASM − 0.072*Ht – 1.349*sex (male = 0, female = 1)	0.853	0.851
Hologic (*n* = 112)		= 10.869 + 1.073*BIA_ASM – 0.089*Ht	0.912	0.910
GE lunar (*n* = 122)		= 3.717 + 0.505*BIA_ASM + 0.082*Wt – 1.879*sex (male = 0, female = 1)	0.894	0.892
Total (*n* = 234)	DXA_ASM/Ht^2^	= 7.041 + 0.889*BIA_ASM/Ht^2^ − 0.039*Ht − 0.563*sex (male = 0, female = 1)	0.690	0.686
Hologic (*n* = 112)		= 1.634 + 1.077*BIA_ASM/Ht^2^ – 0.019*Ht	0.825	0.822
GE lunar (*n* = 122)		= 8.160 + 0.566*BIA_ASM/Ht^2^ − 0.045*Ht + 0.032*Wt − 0.747*sex (male = 0, female = 1)	0.768	0.760
Total (*n* = 234)	DXA_ASM/BMI	= 0.255 + 0.716*BIA_ASM/BMI − 0.001*Wt − 0.061*sex (male = 0, female = 1)	0.841	0.838
Hologic (*n* = 112)		= 0.752 + 1.158*BIA_ASM/BMI – 0.007*Ht + 0.002*Wt	0.908	0.906
GE lunar (*n* = 122)		= 0.259 + 0.655*BIA_ASM/BMI − 0.076*sex (male = 0, female = 1)	0.872	0.870
Total (*n* = 234)	DXA_ASM/Wt	= 0.269 + 0.828*BIA_ASM/Wt − 0.001*Ht − 0.022*sex (male = 0, female = 1)	0.648	0.644
Hologic (*n* = 112)		= 0.475 + 0.019*BIA_ASM – 0.002*Ht – 0.005*Wt	0.796	0.791
GE lunar (*n* = 122)		= 0.182 + 0.517*BIA_ASM/Wt − 0.001*Wt – 0.031*sex (male = 0, female = 1)	0.691	0.683

By multiple regression analyses

ASM, appendicular skeletal muscle mass; DXA, dual-energy X-ray absorptiometry; BIA, Bioelectrical impedance analysis; Ht, height; Wt, weight; BMI, body mass index

### Factors influencing the discrepancy in muscle mass measurements between BIA and DXA

Multivariable linear regression analysis, which included age, sex, height, weight, BMI, and the type of DXA machine as variables, identified factors influencing discrepancies in muscle mass measurements between BIA and DXA. Sex emerged as a significant predictor of ASM and its indices (ASM/ht², ASM/BMI, and ASM/wt) (standardized β = 0.014–0.814; *p* = 0.001), with women showing greater discrepancies between BIA and DXA measurements compared to men. Height also contributed to these discrepancies (standardized β = 0.001–0.103; *p* < 0.001), indicating that taller individuals tend to exhibit larger discrepancies. The type of DXA machine used also influenced the results (standardized β = −0.024 to − 1.390; *p* < 0.001), suggesting that different DXA machines may yield varying ASM results when compared to BIA (Table 4).

**Table 4:** Factors influencing the discrepancy in measurements between BIA and DXA

Outcome/Independent predictor	Standardized (ß)	*p*-value	Adjusted *R*^2^
ASM difference			0.446
Sex	0.814	0.001	
Height (cm)	0.103	< 0.001	
DXA machine	-1.390	< 0.001	
ASM/Ht2 difference			0.424
Sex	0.361	< 0.001	
Height	0.037	< 0.001	
DXA machine	-0.574	< 0.001	
ASM/BMI difference			0.409
Sex	0.035	0.001	
Height	0.004	< 0.001	
DXA machine	-0.057	< 0.001	
ASM/Wt difference			0.380
Sex	0.014	0.001	
Height	0.001	< 0.001	
DXA machine	-0.024	< 0.001	

Multivariable linear regression analysis adjusting for age, sex, height, weight, BMI, and DXA machine. For the DXA machine variable, Hologic is coded as 0 and GE Lunar as 1

BIA, Bioelectrical impedance analysis; DXA, dual-energy X-ray absorptiometry; ASM, appendicular skeletal muscle mass; Ht, height; Wt, weight

### Bland–Altman plots for agreement between BIA and DXA devices

The Bland–Altman plots, as shown in Fig S2, were used to analyze the agreement between muscle mass measurements from BIA and the two different DXA devices. The assessment was conducted separately for each device and across various indices, with the results presented in four subfigures (Fig S2a-d).

The analysis revealed discrepancies in measurement outcomes between the Hologic Horizon and GE Lunar devices when compared to BIA results. Specifically, the Hologic Horizon displayed a mean difference of − 2.10 in ASM from BIA, indicating a notable variance, while the GE Lunar showed a much smaller mean difference of − 0.47 (Fig S2a). Differences in ASM/ht² were also evaluated, revealing that the mean values varied between the DXA devices (Fig S2b). Measurements of ASM/BMI (Fig S2c) and ASM/wt (Fig S2d) exhibited minimal mean differences across devices, demonstrating overall consistency. However, it is crucial to note that despite these discrepancies, the majority of values consistently remained within the 95% confidence interval, suggesting that such differences are within clinically acceptable limits. Variability between DXA devices when measuring similar parameters was observed in some values, further illustrating the inherent measurement differences across these technologies.

## Discussion

Diagnosing sarcopenia in older adults, particularly those with MetS, poses complex challenges that require accurate and reliable measurement techniques. Our multicenter prospective cohort study sought to address this challenge by conducting a comparative analysis of muscle mass assessments performed using BIA and leading DXA devices. The primary objective was to evaluate the concordance between these two predominant methods of muscle mass assessment and to identify any discrepancies. In response to these discrepancies, we aimed to develop equipment-specific equations to mitigate the differences and enhance the precision of sarcopenia diagnoses.

In our study, BIA consistently overestimated muscle mass compared to DXA devices, a finding consistent with previous studies [17, 21–23]. The degree of overestimation varied significantly among different DXA devices. Similarly, Park et al. [14] found substantial differences in lean mass measurements among 40 patients when using different DXA machines. These significant discrepancies in measurements, not only between BIA and DXA but also across various DXA devices, could complicate the diagnosis of sarcopenia in clinical settings. This underscores the importance of meticulous calibration and careful selection of diagnostic tools to enhance the reliability of sarcopenia diagnoses and prevent complications in patient care.

Our findings demonstrate that while sex significantly influences the discrepancies between BIA and DXA measurements overall, the impact of sex varies by DXA device. For the GE Lunar DXA, sex significantly affects the prediction equations, indicating a sensitivity to sex-based physiological differences that influence muscle mass measurements. In contrast, the Hologic DXA equations do not include sex as a variable, suggesting either a lesser sensitivity to these differences or a different calibration approach.

Previous research has similarly demonstrated discrepancies in ASM and ASMI values measured by BIA and DXA in sex [24, 25], with differences typically more pronounced in women. Our study aligns with these findings, showing that women generally exhibit greater discrepancies between BIA and DXA measurements, although the degree of this discrepancy is influenced by the specific DXA device used. In general, BIA’s body composition assessments are more susceptible to factors like body hydration status, owing to its reliance on different biophysical principles, particularly those involving body water [26, 27]. Additionally, anthropometric variables such as circumference, length, and volume can differentially impact estimates by DXA and BIA, given the distinct ways in which these variables interact with X-ray imaging versus the three-dimensional flow of electrical currents [28]. While these diagnostic differences have often been attributed to the physiological characteristics of women, such as body fat distribution and total body water content [29, 30], our findings suggest that the influence of these characteristics may vary depending on the DXA device used.

Furthermore, our analysis revealed that height significantly impacts the discrepancies observed between BIA and DXA measurements of ASM and ASMI values, with taller individuals exhibiting larger variances. This finding aligns with previous research identifying height as a predictor of whole-body differences in fat mass and lean soft tissue [28]. Supporting evidence is provided by Wang et al. [31], who studied 57 patients with type 2 and 3 spinal muscular atrophy and found that height significantly influenced muscle mass measurements obtained via both BIA and DXA methods. They reported that discrepancies increased with patient height, suggesting that inherent differences in body structure and composition accoridng to height—such as a larger skeletal frame and potentially more muscle mass in taller individuals—can affect these measurements. The extended path for electrical signals in BIA measurements for taller individuals may also contribute to these differences, underscoring the need to carefully consider height as a variable in sarcopenia diagnosis.

In our study, we developed equations to address and clarify the discrepancies between different diagnostic tests and devices. These equations, derived from DXA measurements, demonstrated high adjusted R² values (0.851 for the entire population, 0.892–0.910 for device-specific models), indicating strong predictive power and consistency with prediction rates from previous studies [17, 32–34]. While DXA measurements are generally more accurate for assessing muscle mass than BIA, significant variability exists among different DXA devices. By tailoring BIA calculations to account for the specific DXA device used, we can effectively manage this variability and achieve more reliable muscle mass predictions across various DXA machines. This finding underscores the potential of BIA as a viable, less-invasive alternative for routine sarcopenia assessment in clinical settings, potentially simplifying the diagnostic process and improving patient care.

This study is notable for its comparative analysis of muscle mass assessments between BIA and DXA in older adults with MetS. MetS and sarcopenia interact through various mechanisms, significantly increasing health risks for patients with both conditions compared to those with either condition alone [35]. Both MetS and sarcopenia adversely impact quality of life and contribute to increased frailty, weakness, dependence, and morbidity and mortality rates [36]. A Korean epidemiological study found that out of 3,305 individuals with MetS, 739 (22.4%) also had sarcopenia [37]. These findings highlight the importance of early and accurate diagnosis and treatment of these conditions. Prompt intervention is essential not only for improving clinical outcomes but also for enhancing overall quality of life and reducing the risk of severe health complications associated with the coexistence of MetS and sarcopenia. Additionally, our comparative analysis of different DXA devices underscores the significant contributions of our findings to the field of sarcopenia research. Understanding the differences in measurement and equipment, and applying equations to address these disparities, offers a viable alternative for accurate diagnosis.

While our study offers significant insights into the assessment of sarcopenia in older adults with MetS, it has some limitations. First, the study focused on a specific group: individuals with MetS suspected of sarcopenia who were tested in large hospitals. This selection criterion means our findings are particularly relevant to this group and may not be fully generalizable to all older adults or those with different health profiles. Additionally, the study’s hospital-based setting might have influenced the results, as patients in such environments may have different characteristics or health conditions compared to those in community-based settings or smaller medical facilities. Another important limitation is our study’s relatively small sample size, which restricts the universal applicability of the proposed predictive equations. Future research should investigate a broader range of patients, including those not limited to sarcopenia suspicion, to validate and refine the predictive equations developed in our study. Furthermore, conducting studies across various healthcare settings, such as smaller clinics or community-based environments, would help assess the broader applicability of our findings.

Lastly, as discrepancies between BIA and DXA measurements may be modified by factors such as sarcopenic obesity, further exploration into patient profiles that minimize these discrepancies is warranted. Standardizing measurements across devices is essential, and longitudinal studies examining the impact of measurement differences on clinical outcomes in older adults will provide further valuable insights into improving sarcopenia diagnosis and management.

## Conclusions

In conclusion, our study highlights the discrepancies between muscle mass measurements obtained by BIA and different DXA devices in older adults with MetS. By developing device-specific equations, this research provides a practical approach to align BIA results with DXA measurements, supporting more accurate assessments of muscle mass in clinical settings. The observed variation in prediction formulas underscores the need for standardized diagnostic methods across different measurement devices, which is crucial for advancing the accuracy and consistency of sarcopenia diagnosis, ultimately enhancing clinical outcomes for older adults with MetS.

## Electronic supplementary material

Below is the link to the electronic supplementary material.

Supplementary Material 1

Supplementary Material 2

## Data Availability

No datasets were generated or analysed during the current study.
