# Supplementary material for: Comparison of bioelectrical impedance analysis and dual-energy X-ray absorptiometry for the diagnosis of sarcopenia in the older adults with metabolic syndrome: equipment-specific equation development
Source: Aging Clin Exp Res. 2024 Dec 27;37(1):12. doi: 10.1007/s40520-024-02898-1 (PMC11671549; doi:10.1007/s40520-024-02898-1)

Fig. S1 Bland-Altman plot comparing ASM and ASM indices measured by BIA and DXA

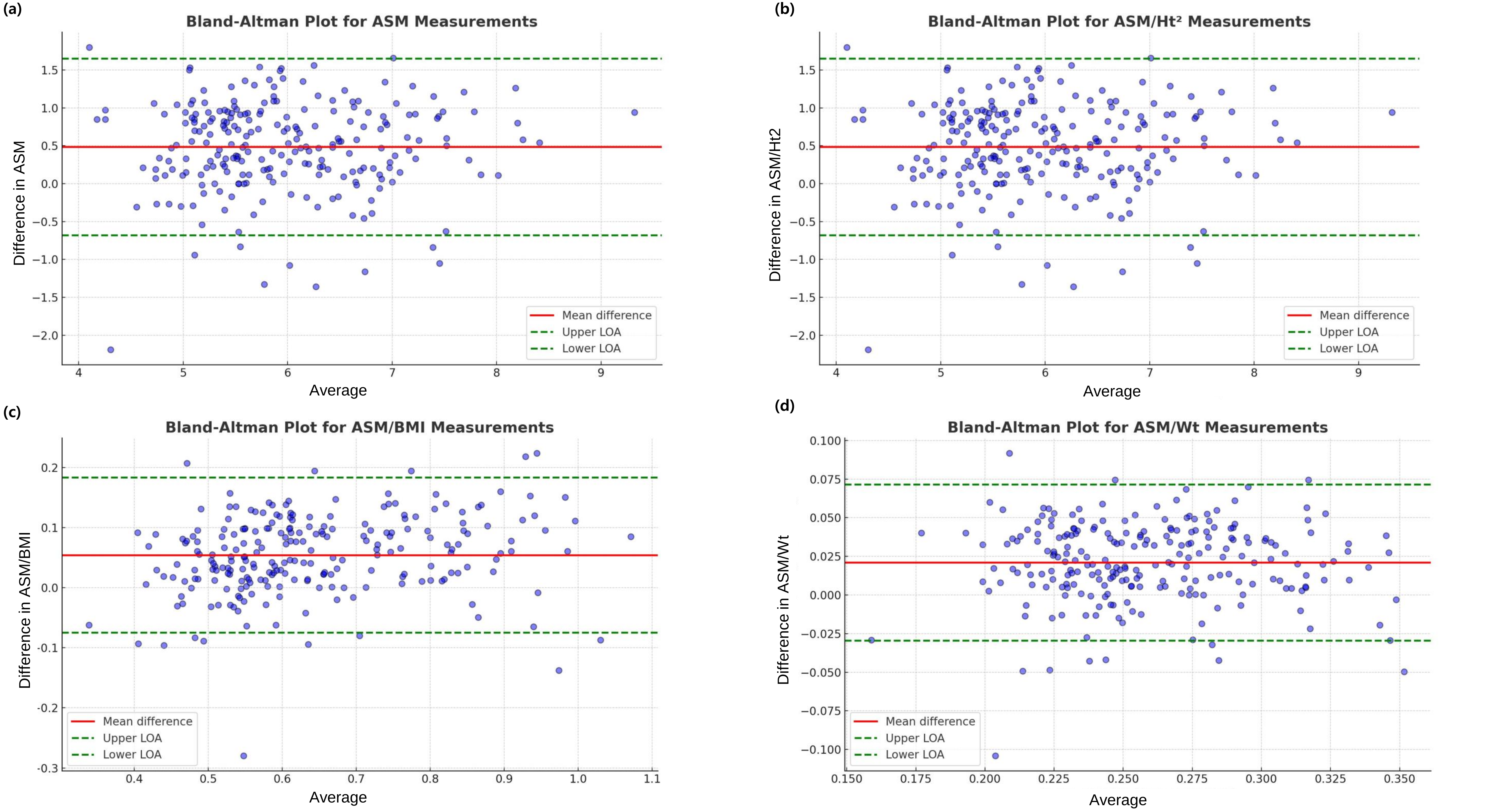

Fig. S2 Bland-Altman plot comparing ASM and ASM indices measured by BIA and each DXA device

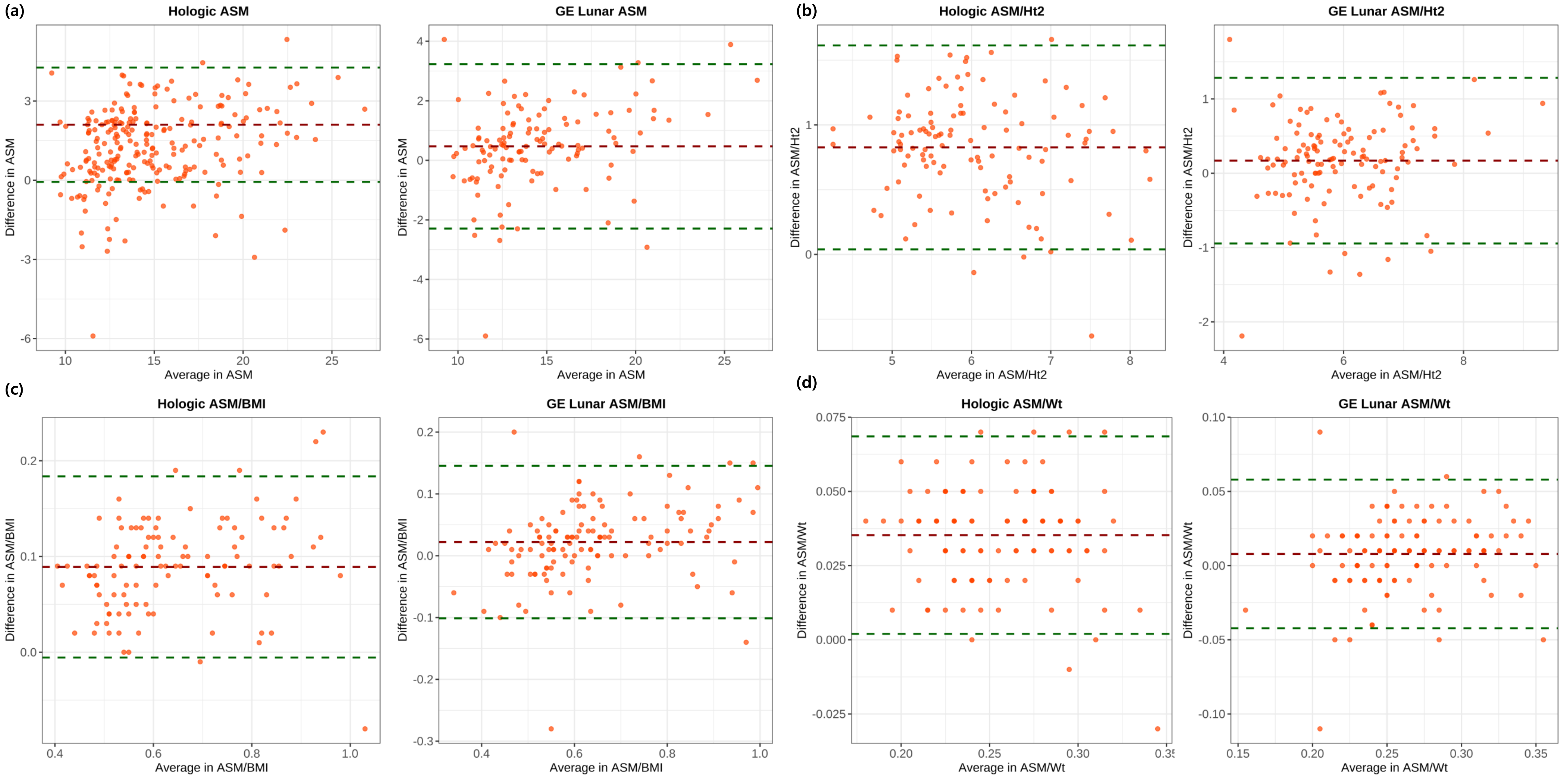

Supplement: Supplementary file 1 — Supplementary Material 1 [file 40520_2024_2898_MOESM1_ESM.pdf]
