# Supplementary material for: Comparison of bioelectrical impedance analysis and dual-energy X-ray absorptiometry for the diagnosis of sarcopenia in the older adults with metabolic syndrome: equipment-specific equation development
Source: Aging Clin Exp Res. 2024 Dec 27;37(1):12. doi: 10.1007/s40520-024-02898-1 (PMC11671549; doi:10.1007/s40520-024-02898-1)
Supplement: Supplementary file 2 — Supplementary Material 2 [file 40520_2024_2898_MOESM2_ESM.docx]

**Fig. S1** Bland-Altman plot comparing ASM and ASM indices measured between BIA and DXA

(A) ASM (B) ASM/height^2^ (C) ASM/BMI (D) ASM/weight

Top and bottom reference lines indicate 95% confidence interval. Abbrevations: ASM, appendicular skeletal muscle mass; BIA, bioimpedance analysis; DXA, dual-energy X-ray absorptionmetry

**Fig. S2** Bland-Altman plot comparing ASM and ASM indices measured between BIA and two types of DXA devices

(A) ASM (B) ASM/height^2^ (C) ASM/BMI (D) ASM/weight

Top and bottom reference lines indicate 95% confidence interval. Abbrevations: ASM, appendicular skeletal muscle mass; BIA, bioimpedance analysis; DXA, dual-energy X-ray absorptionmetry
